# An Ultra-Broadband Conductor-Backed Coplanar Waveguide with Sine Edges

**DOI:** 10.3390/s24206640

**Published:** 2024-10-15

**Authors:** Tingting Xie, Pengwei Gong, Xiaohe Cheng, Tao Xiu, Yuan Yao

**Affiliations:** 1 Beijing Institute of Radio Metrology and Measurement, Beijing 100854, China; tingtingx96@163.com; 2 School of Electronic Engineering, Beijing University of Posts and Telecommunications, Beijing 100876, China

**Keywords:** conductor-backed coplanar waveguide with sine edges (CBCPW-SE), low loss, periodic sine stubs

## Abstract

In this paper, a conductor-backed coplanar waveguide with sine edges (CBCPW-SE), consisting of a conductor-backed coplanar with periodic sine edges supported by a dielectric substrate, is proposed as a promising new transmission line (TL) at millimeter-wave and terahertz frequencies. This configuration offers a distinct advantage by maintaining a constant input impedance of 50 Ω across a broad frequency spectrum, eliminating the necessity for any additional impedance matching transitions, thereby enhancing the overall efficiency and simplicity of the transmission system. To verify the design, the CBCPW-SE was fabricated and measured. The measurement results demonstrate that, from 10 MHz to 100 GHz, the insertion loss is less than 0.1 dB/mm and the reflection coefficient is better than −10 dB. The measured S21 (dB) for a 50 mm-long CBCPW-SE section is less than −5 dB from 10 MHz to 100 GHz. The measured group delay per unit length of the trace is 3.4–5 ps, ranging from 10 MHz to 100 GHz. Moreover, the measured group velocity dispersion (GVD) approaches zero, signifying the minimal temporal spreading of the signal components, a crucial aspect for maintaining signal integrity and facilitating high-speed data transmission.

## 1. Introduction

Transmission lines serve as the medium for guiding the propagation of electromagnetic waves and constitute a vital component in the fabrication of devices and circuits. Their characteristics directly influence the bandwidth, dimensions, and functionality of these systems. Transmission lines have great potential for ultra-wideband interconnections, including chip-to-chip interconnects, picosecond-scale transmissions, and ultrafast data transfers, especially in the context of advances in ultra-high-speed systems. Transmission lines (TLs) are paramount to communication systems, ensuring superior signal integrity, particularly for applications spanning the frequency range from direct current (DC) to terahertz (THz) [1]. Indeed, TLs underpin all high-frequency integrated circuits, with their performance metrics and cost efficiencies being inherently constrained by the properties of these lines. Consequently, the selection of low-cost, low-loss, and broadband TLs as core modules has become increasingly critical. Thus, the realization of low-loss, broadband, and miniaturized TLs operating at millimeter-wave or terahertz frequencies has emerged as a pivotal research focus.

There are various types of planar transmission lines, including the microstrip line (ML), ground coplanar waveguide (GCPW), conductor-backed CPW (CBCPW), substrate-integrated waveguide (SIW), and mode-selective transmission lines. According to the transmission mode, planar TLs can be divided into three types: TE/TM, TEM, and a mode-selective TL (MSTL). Conventional substrate-integrated waveguides (SIWs), leveraging their low-loss advantage at high frequencies through the transmission of the TE10 mode, have been extensively employed in device designs and facilitate seamless integration with other components. However, the fundamental mode bandwidth of SIWs is inherently constrained by the cutoff frequency and the emergence of higher-order modes (such as the TE20 and TE01 modes), limiting their achievable bandwidth to approximately 40%, which is inadequate for high-speed systems [2,3]. Commonly, planar TLs, including the GCPW, ML, and CBCPW, support pure TEM waves without a cutoff frequency but also encounter the problems of high loss, dispersion, and high-order modes at a high frequency [4,5,6]. The loss of higher-order modes and radiation loss cause the CBCPW to experience energy leakage. The GCPW suppresses the higher-order modes and radiation, reduces energy leakage, and thus widens the CBCPW bandwidth [7]. The GCPW supports circuit transmission up to 110 GHz, yet necessitates the use of two rows of metal vias, thereby augmenting the complexity of the fabrication process. While the ML and CBCPW exhibit commendable transmission performance at lower frequencies, their performance degrades significantly in the millimeter-wave and terahertz frequency bands, manifesting in substantial loss and dispersion phenomena. Consequently, the existing TEM mode transmission lines are not viable for high-frequency broadband systems. Much effort has been devoted to designing an MSTL to achieve low transmission loss and broadband [8,9,10,11,12]. The MSTL supports the dominant TEM mode at a low frequency and the dominant TE10 mode at a high frequency. However, MSTL circuits have challenges because they have two rows of metal sidewall vias. One particular challenge is that the vias on fragile dielectrics are difficult to fabricate for higher frequencies. Metal sidewalls introduce significant inductance and degrade circuit performance over millimeter waves (mm-W) or THz [13].

Conventional TLs and their derivative variants confront formidable challenges, including anomalous resonance phenomena, leaky wave emissions, and intricate fabrication processes in pursuit of an ultra-high-speed interconnection and picosecond-scale transmission within chip circuits. As the operational frequency escalates, the analysis and design of ultra-broadband TLs become increasingly intricate, owing to significant loss mechanisms, pronounced dispersion effects, and the emergence of higher-order modes. Consequently, the realization of low-loss, broadband, and miniaturized TLs operating at mm-W or THz frequencies has garnered immense significance, underscoring the need for innovative solutions. Hence, there is a pressing demand to develop an alternative transmission line architecture that not only addresses the aforementioned challenges but also offers advantages in terms of simplified fabrication procedures, broad operational bandwidth, and minimal loss characteristics, thereby catering to the rigorous requirements of mm-W and THz applications. Such a transmission line would represent a significant advancement in the field, enabling the efficient and reliable transmission of ultra-high-speed signals within chip circuits.

In this paper, the ultra-broadband CBCPW-SE with low loss and low dispersion is presented for the first time. Periodic sine stubs have stopband characteristics. To verify the design, a prototype was fabricated and measured. The measured insertion loss was less than 0.1 dB/mm, and the measured reflection coefficient was better than −10 dB from DC to 110 GHz, which agrees well with the simulations. The measured group delay per unit length of the trace was 3.4–5 ps, ranging from 10 MHz to 100 GHz, and the measured group velocity dispersion (GVD) was near-zero. In the second section of this paper, we delineate the structural prototype and its underlying operational principles. Subsequently, the third section delves into the fabrication and measuring procedures, followed by a comprehensive analysis of the results. Lastly, the fourth section encapsulates the overall conclusion of this paper.

## 2. Analysis of the CBCPW-SE

### 2.1. Geometry of the Structure

**Figure 1:**
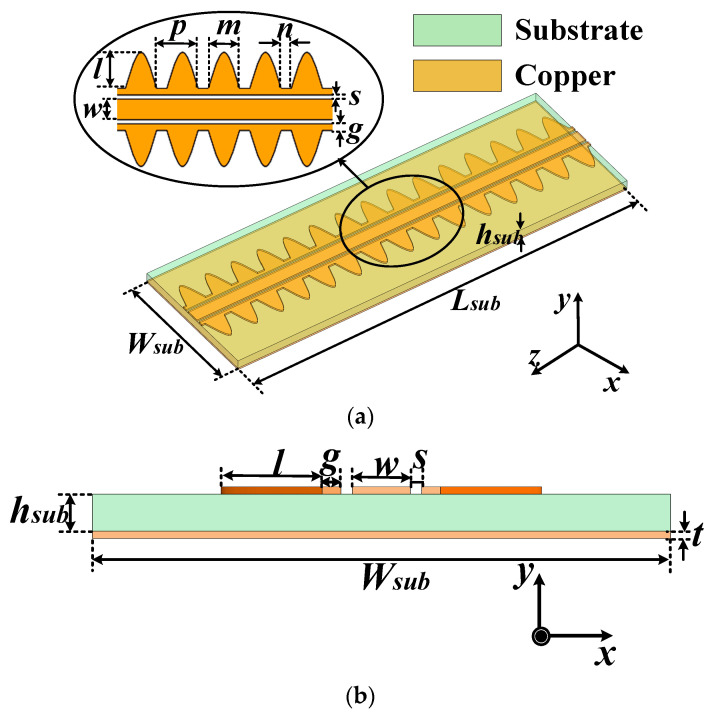
Configuration of the proposed transmission line: (**a**) 3D view; and (**b**) cross-sectional view.

Three-dimensional and cross-sectional views of the proposed CBCPW-SE are presented in Figure 1. The CBCPW-SE consists of the CBCPW with periodic sine stubs lined up at the edges. The sine edges prevent wave leakage like an equivalent “metal wall”. A Rogers 5880 (Rogers Corporation, Chandler, Arizona, USA) printed circuit board (PCB) laminate with a thickness of 0.254 mm and a dielectric constant of 2.2 is utilized to realize the design. The width of the CBCPW center metallic strip and slots are w and *s*, respectively. The periodic sine stubs with the width of *m*, depth of *l*, and spacing of *p* are selected as the sine units. The overall width and length of the CBCPW-SE are *Wsub* and *Lsub*, respectively. The dimensions of the CBCPW-SE are summarized in Table 1.

### 2.2. Working Principle

**Figure 2:**
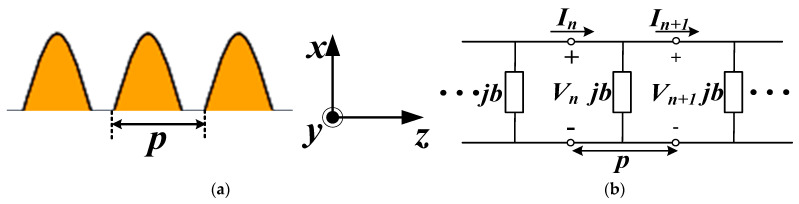
Geometries and equivalent networks of period sine structure: (**a**) periodic structure; and (**b**) equivalent circuit model.

Figure 2a,b show the configuration and equivalent model of the period sine structure, respectively. The edge of the CBCPW-SE comprises the infinite repetition of the period sine cell, in which the admittance jb is separated by a distance d on the *z*-axis [14,15,16]. The performance of the unit cell can be expressed by its equivalent ABCD matrix [14]. Therefore, the input and output of the n-th unit cell are expressed by the following

(1) $$\left[\begin{array}{l} V_{n}\\ {Ι}_{n} \end{array}\right]=\left[\begin{array}{l} A\\ C \end{array}\right.\left.\begin{array}{l} B\\ D \end{array}\right]\left[\begin{array}{l} V_{n+1}\\ I_{n+1} \end{array}\right]$$

where

A=cosθ−b/2sinθ, B=j(sinθ+b/2cosθ−b/2)

C=j(sinθ+b/2cosθ−b/2), D=cosθ−b/2sinθ

From [14], the requirement for no wave propagation in the periodic sine structure in the form of infinite cascaded unit cells is as follows:

(2) $$\cos\gamma d=\left|\cos\theta -b/2\sin\theta \right|=0.5\left|A+D\right|\geq 1$$

When the condition of Equation (2) is satisfied, the periodic sine structure cannot propagate the wave, defined as a stopband.

In the above, *b* is the admittance, *θ* = *kd* is the electrical length, *k* is the propagation constant, and *p* is the period. The complex propagation constant is γ.

**Figure 3:**
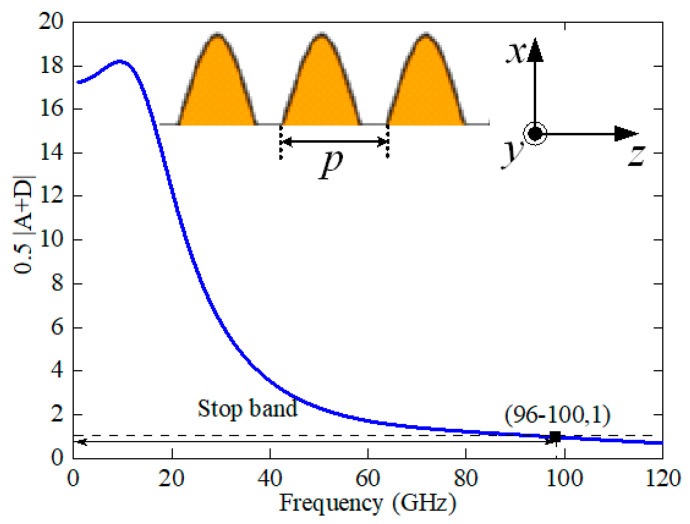
Method analysis. Simulated result of |0.5(A + D)|, where A and D are parameters of the ABCD matrix of the equivalent stub circuit in Figure 2.

The model is shown in Figure 2 for analyzing the sine unit cell with *p* = 0.4 mm, *m* = 0.6 mm, *n* = 0.2 mm, and *l* = 0.7 mm. The ABCD matrix of the unit cell can be extracted from the HFSS simulated data. The simulated value of 0.5|A + D| is shown in Figure 3, indicating that the stopband is from DC to 96 GHz (where 0.5|A + D| ≥ 1). This corroborates the bandstop characteristics exhibited by the sine unit cell of the transmission line within the DC-96 GHz frequency range, effectively mitigating the leakage of waves outwards.

**Figure 4:**
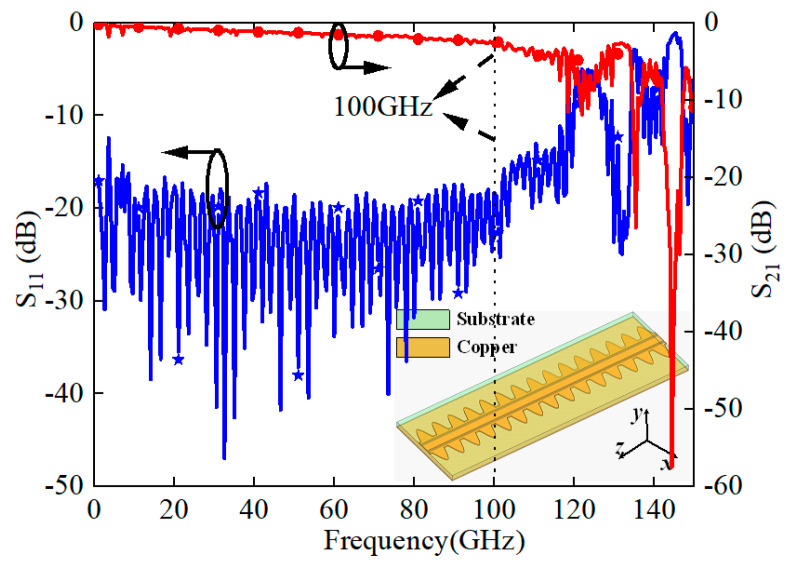
S parameters. (The red line represents S_21_ and the blue line represents S_11_).

The structure was solved for the *S* parameters by the HFSS, which has an enormous insertion loss (S_21_) above 100 GHz, as shown in Figure 4. This observation aligns with the conclusions drawn from Figure 3, underscoring the bandstop characteristics inherent to the sine unit cell of the transmission line.

**Figure 5:**
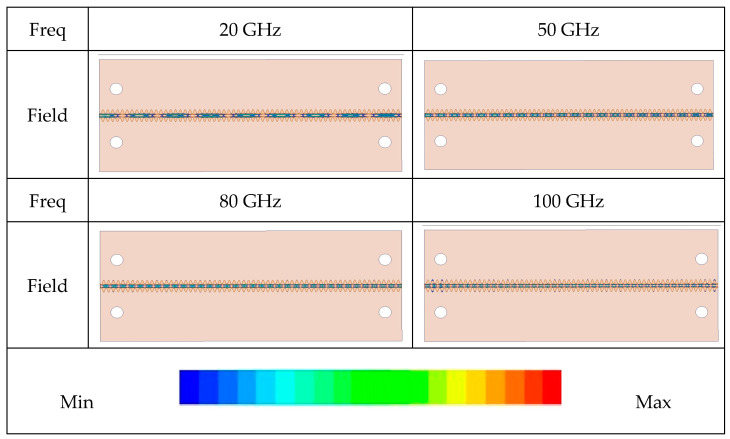
The electric field distribution at different frequencies.

The electric field distribution from 20 GHz to 100 GHz is shown in Figure 5, demonstrating that the field is well constrained in the middle of the CBCPW-SE as the frequency increases. In addition, it can propagate the quasi-TEM mode. Therefore, the periodic sine of the edges blocks wave leakage, which is like an equivalent “metal wall”. The field distribution of the transmission line is consistent with the aforementioned bandstop characteristics inherent to its sine unit cell.

**Figure 6:**
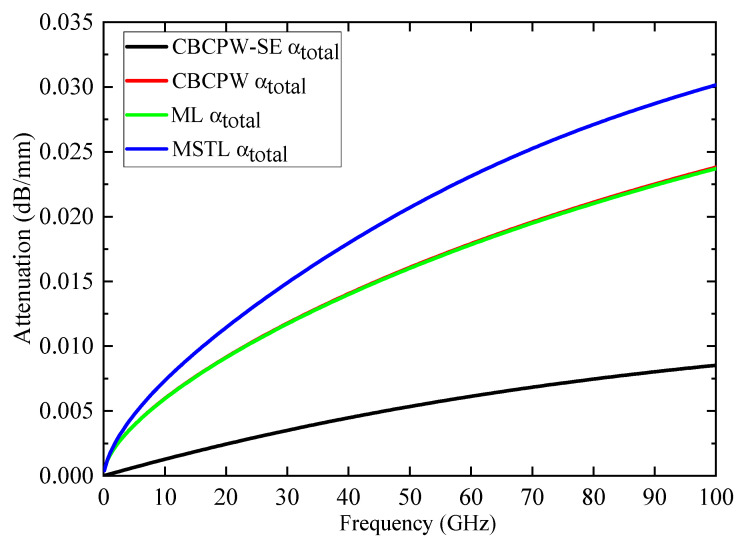
Comparison of the attenuation constants of the proposed CBCPW-SE, CBCPW, ML, and MSTL.

To estimate the loss of the proposed CBCPW-SE, the simulated attenuation coefficients of total loss $\alpha_{total}$, compared with the simulation results of the CBCPW, ML, and MSTL, are given in Figure 6. The thickness, width, and dielectric substrate of these four transmission lines are the same in the simulation. It can be observed that, as the frequency increases, the attenuation constant of the proposed CBCPW-SE is significantly lower than that of the CBCPW, ML, and MSTL, which proves that the CBCPW-SE has extremely low losses.

## 3. Experimental Results and Discussion

### 3.1. Fabrication and Measurement Process

**Figure 7:**
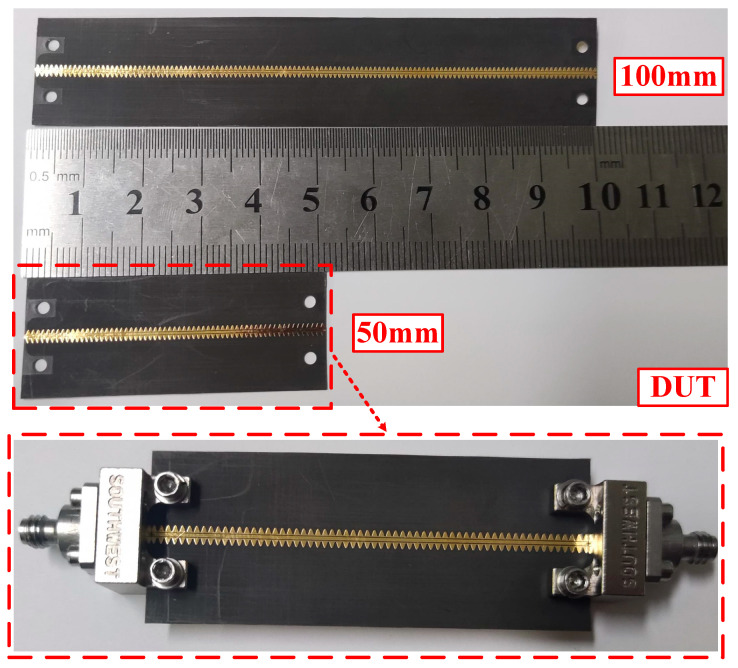
Photographs of the fabricated devices. Two lengths of CBCPW-SE: 50 mm and 100 mm; and the measurement connectors.

The photographs depicting the envisioned TL and the corresponding measurement connectors are presented in Figure 7 in a detailed visualization. The CBCPW-SE, featuring two distinct lengths of 50 millimeters (mm) and 100 mm, was meticulously fabricated employing advanced printed circuit board (PCB) technology, ensuring precision and conformity with the design specifications.

**Figure 8:**
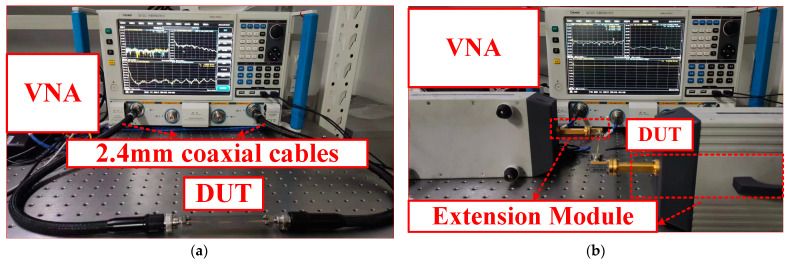
Photograph of the measurement process: (**a**) devices under test (DUTs) from 10 MHz to 50 GHz; and (**b**) devices under test (DUTs) from 50 to 75 GHz and from 75 to 100 GHz.

To further verify the performance of the proposed CBCPW-SE experimentally, we measured the fabricated CBCPW-SE sample shown in Figure 8 using the Ceyear 3672D vector (Ceyear China, Qingdao, China) network analyzer (VNA). The operating frequency band ranged from 10 MHz to 100 GHz. For the 10 MHz–50 GHz measurement, two 2.4 mm coaxial cables connected to the VNA were fixed to the device. The VNA was used along with 50–75 GHz and 75–100 GHz extending modules to perform measurements from 50 to 100 GHz. The transitions, including at the waveguide to coaxial connectors and the 1 mm end launch insertion loss, cannot be eliminated by calibration, so we fabricated and measured two lengths (50 mm and 100 mm) to obtain a differential insertion loss (with a measurement of |100–50 mm|). Differential calculations were required to measure the insertion loss and group delay (with a measurement of |100–50 mm|).

### 3.2. Simulation and Measurement Results

**Figure 9:**
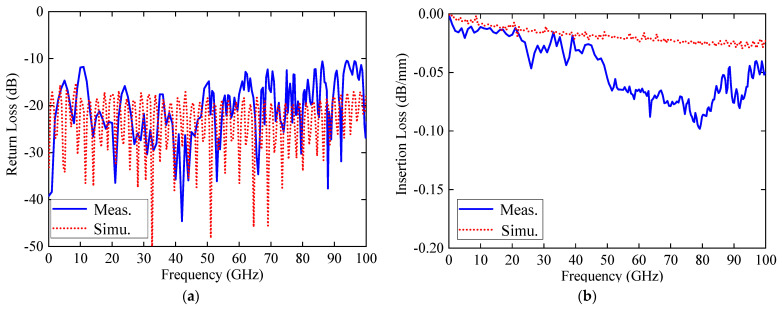
Simulated and measured performance of the CBCPW-SE: (**a**) return loss; and (**b**) insertion loss.

The simulated and measured reflection and transmission coefficients are displayed in Figure 9. The Huray model with a Hall–Huray surface ratio of 0.5 and a nodule radius of 1.8 μm was employed to simulate the influence of surface roughness [17]. It showed that both the simulated and measured S_11_ are less than −10 dB in the frequency range from 10 MHz to 100 GHz. Furthermore, both the simulated and measured S_21_ are larger than −5 dB with a length of 50 mm (with a measurement of |100–50 mm|), and a measured insertion loss lower than 0.1 dB/mm from 10 MHz to 100 GHz is achieved, as shown in Figure 9b. The small discrepancies between the simulated and measured insertion losses across the entire frequency range can primarily be attributed to measurement and fabrication errors. Furthermore, the inevitable introduction of the metal’s surface roughness during the PCB fabrication may also contribute to these differences. Return loss (S_11_) is included with various transition connectors during the measuring process. The deterioration of S_11_ is mainly due to the loss from the transition connectors.

**Figure 10:**
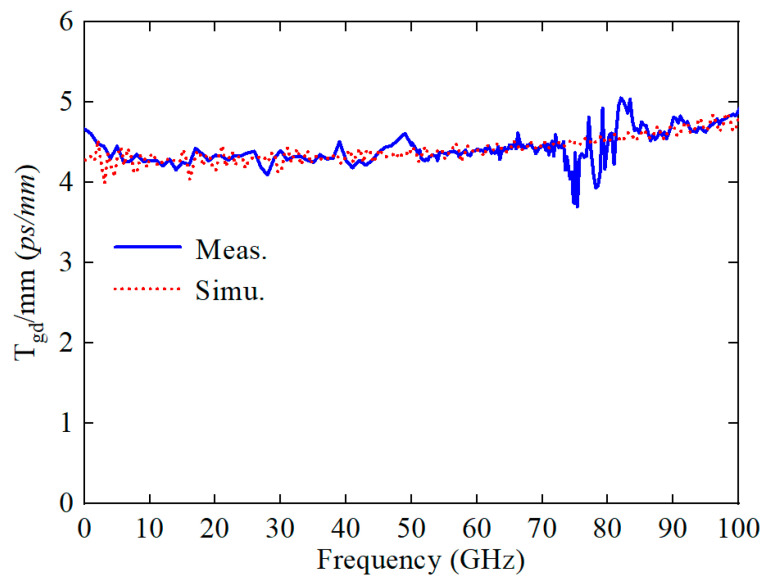
Simulated and measured unit length group delay.

Figure 10 shows that the CBCPW-SE supports wave propagation with a low and flat group delay (*T_gd_*). Specifically, the group delay per unit length is 3.4–5 ps, ranging from 10 MHz to 100 GHz.

**Figure 11:**
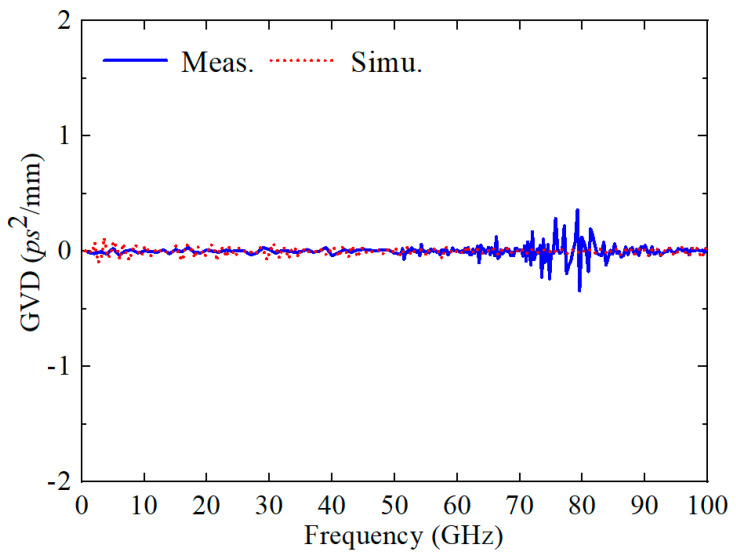
Simulated and measured GVD.

To delve deeper into the dispersion properties of the CBCPW-SE within the broadband regime, we undertook a rigorous analysis by computing the group velocity dispersion (GVD), as depicted in Figure 11. The GVD values, which are proximal to zero across the entire frequency band, underscore the exceptional low dispersion nature of the structure. This feature is crucial for preserving signal integrity and minimizing distortion in high-speed signal transmission systems. In general, the experimental outcomes align well with the predictions derived from the simulations, reinforcing the validity of our design and analysis. However, a marginal discrepancy in magnitude can be attributed primarily to the inherent limitations in the measurement process and the fabrication tolerances, which are common challenges encountered in the realization of microwave and millimeter-wave components. Nonetheless, these findings underscore the robust performance and potential of the CBCPW-SE for applications requiring precise control over wave propagation characteristics.

In a comprehensive analysis, Table 2 presents a comparative evaluation of the transmission characteristics exhibited by diverse transmission line configurations. Notably, the CBCPW-SE stands out as a promising candidate, offering the dual advantages of broadband operation and reduced loss compared to its counterparts, namely, the SIW, CBCPW, and GCPW. Specifically, the measured insertion loss of the CBCPW-SE remains below 0.1 dB/mm from 10 MHz to 100 GHz, underscoring its superiority for broadband applications. Furthermore, in contrast to the SIW, GCPW, and MSTL, the CBCPW-SE design eliminates the need for vias in its fabrication process, thereby simplifying the manufacturing procedure. Nevertheless, as the operational frequency escalates into the THz regime, the vias dimensions diminish significantly, posing challenges for their realization using conventional PCB technology. This limitation highlights the need for innovative fabrication techniques or alternative designs to fully harness the potential of the CBCPW-SE in the THz domain.

## 4. Conclusions

This paper presents the CBCPW-SE, which is composed of the CBCPW and periodic sine stubs at the edges. The sine edges act as a kind of “metal wall”, preventing wave leakage. The proposed CBCPW-SE exhibits several salient features, including ultra-broadband operation, low insertion loss, minimal dispersion, and the absence of vias, thereby enhancing its applicability in high-frequency circuits. Notably, the CBCPW-SE is designed to maintain a characteristic impedance of 50 Ω, eliminating the need for additional impedance-matching transitions. To substantiate the performance claims, a prototype of the CBCPW-SE was fabricated and subjected to rigorous measurements. The experimental results show an impressive insertion loss of less than 0.1 dB/mm over a frequency range of 10 MHz to 100 GHz. Furthermore, the measured group delay per unit length of the transmission line remains within a tight range of 3.4 ps to 5 ps over the entire frequency band, underscoring its low dispersion characteristics. Importantly, the group velocity dispersion (GVD) is measured to be near-zero with a high quality, indicating the minimal distortion of the transmitted signals. These findings collectively validate the exceptional performance of the proposed CBCPW-SE structure, positioning it as a promising candidate for high-speed, low-loss signal transmission in advanced electronic systems.

## Figures and Tables

**Table 1:** Dimensions of the proposed structure (unit: MM).

*w*	*s*	*g*	*l*	*p*	*m*	*n*
0.4	0.08	0.11	0.7	0.8	0.6	0.2
*Wsub*	*Lsub*	*hsub*	*t*			
20	50	0.254	0.018			

**Table 2:** Comparison of the transmission characteristics of the transmission lines.

Ref.	Type	BW (GHz)	Mode	Vias	Measured Insertion Loss (dB/mm)
[18] [19] [20]	SIW	24–40 70–80 75–110	TE10	Yes	0.018 dB/mm (Laminated fused silica) 0.08 dB/mm (Alumina) 0.13 ± 0.02 (ARC)
[8]	CBCPW	DC-60	TEM	No	0.45@60
[6] [4]	GCPW	DC-50 DC-110	TEM	Yes	0.095@50; 0.05@40 GHz; 0.23@110 GHz (Fused silica)
[8] [9]	MSTL	DC-60 DC-110	TEM+ TE10	Yes	0.12@60 (Ceramic PTFE) 0.26@110 (Rogers 6010LM)
This work	CBCPW-SE	DC-100	TEM	No	<0.1 dB/mm (Rogers 5880)

## Data Availability

The complete data are available in this research paper.
